# Investigating the role of serum IL-6 in predicting outcomes of b-cell depleting therapy in multiple sclerosis: a retrospective cohort study

**DOI:** 10.1186/s12883-026-05252-2

**Published:** 2026-08-21

**Authors:** Tristan Kölsche, Ramona Hagler, Niklas Huntemann, Lars Masanneck, Marc Müller, Bela Jesaja Kutsojannis, Joshua M. Boeckers, Patricia Kirschner, Karin Schulze-Bosse, Orhan Aktas, Sven G. Meuth, Tobias Ruck, Marc Pawlitzki

**Affiliations:** 1 Department of Neurology, Medical Faculty University Hospital Düsseldorf, Moorenstraße 5, Düsseldorf, D-40225 Germany; 2 Hasso Plattner Institute, University of Potsdam, Potsdam, Germany; 3 Department of Neurology, Ruhr University Bochum, BG University Hospital Bergmannsheil, Bochum, Germany; 4 Heimer Institute for Muscle Research, BG University Hospital Bergmannsheil, Bochum, Germany; 5 Central Institute for Clinical Chemistry and Laboratory Diagnostics, Medical Faculty, University Hospital Düsseldorf, Heinrich-Heine-University, Düsseldorf, 40225 Germany

**Keywords:** Multiple Sclerosis, Interleukin-6 (IL-6), Ocrelizumab, B-cell Depletion, Primary Progressive Multiple Sclerosis (PPMS), Relapsing-Remitting Multiple Sclerosis (RRMS), Progression Independent of Relapse Activity (PIRA), Biomarker, Serum Neurofilament Light Chain (sNfL), Serum Glial Fibrillary Acidic Protein (sGFAP)

## Abstract

**Background:**

Interleukin-6 (IL-6) plays a pivotal role in autoimmune inflammation through its effects on B-cell differentiation, Th17 expansion, and regulatory T-cell suppression. Given ocrelizumab’s (OCR) mechanism of selective CD20^+^ B-cell depletion, baseline IL-6 levels have been hypothesized to predict disease activity and long-term outcomes in multiple sclerosis (MS). However, the prognostic value of serum IL-6 in OCR-treated patients remains unclear. Therefore, the objective of this study was to evaluate whether baseline and longitudinal serum IL-6 levels can predict clinical and radiological outcomes in OCR-treated MS patients.

**Methods:**

This exploratory, single-center retrospective cohort study included 73 patients with Relapsing-Remitting MS (RRMS, *n* = 30) or primary progressive MS (PPMS, *n* = 43) who initiated OCR at University Hospital Düsseldorf between 2018 and 2025. Baseline serum IL-6 was compared with 86 healthy controls (HC) and correlated with clinical, radiological, and biomarker outcomes over 24 months. Clinical endpoints included confirmed progression independent of relapse activity (PIRA), and relapse-associated worsening (RAW), alongside MRI activity and Serum Neurofilament Light Chain (sNfL) and Serum Glial Fibrillary Acidic Protein (sGFAP) levels. Between-group comparisons used Welch’s t-test or Wilcoxon rank-sum test, paired longitudinal comparisons used paired t-tests or Wilcoxon signed-rank tests, and associations between baseline biomarkers and outcomes were evaluated using Cox regression, multivariable linear or logistic regression, and rank-based linear models for IL-6.

**Results:**

Baseline serum IL-6 levels showed no significant differences between the overall MS cohort and HC (*p* = 0.178), nor between RRMS and PPMS subgroups (*p* = 0.024; adjusted post-hoc *p* > 0.05). No baseline biomarker, including IL-6 (HR 1.83; 95% CI 0.73–4.61; *p* = 0.989), sNfL, and sGFAP predicted disease activity. Longitudinal analysis under OCR revealed largely stable IL-6 concentrations but patients maintaining No Evidence of Disease Activity-3 (NEDA-3) showed 50% reduction in IL-6 at 12 months (mean change − 1.30 pg/mL, *p* = 0.0318), whereas those with loss of NEDA-3 remained stable (mean change 0.09 pg/mL).

**Conclusion:**

Baseline serum IL-6 alone is insufficient to predict clinical or radiological outcomes in OCR-treated MS patients. However, the significant longitudinal decline specifically in stable patients suggests that IL-6 dynamics, rather than static baseline measures, may better reflect sustained therapeutic response. These findings underscore the limited utility of serum IL-6 alone as a biomarker and support further exploration of longitudinal, multiparametric approaches.

**Supplementary Information:**

The online version contains supplementary material available at 10.1186/s12883-026-05252-2.

## Introduction

Multiple sclerosis (MS) is a chronic demyelinating and inflammatory disease of the central nervous system in which both T-cells and B-cells play critical roles in driving tissue damage [1]. MS often leads to progressive functional impairment, requiring not only pharmacological treatment but also multidisciplinary management including rehabilitation strategies, assistive devices, and long-term socio-economic support [2]. Identifying reliable biomarkers to predict treatment response is critical to guide these long-term management decisions. Among pro-inflammatory mediators, interleukin-6 (IL-6) has emerged as a particularly interesting cytokine due to its multifaceted effects on immune cell differentiation and function. IL-6 promotes B-cell differentiation and immunoglobulin production, potentially contributing to MS pathology. It also facilitates Th17 cell expansion while inhibiting regulatory T-cell development, fostering a pro-inflammatory environment that may exacerbate neuroinflammation and demyelination [3]. Consequently, IL-6 is considered a key driver of autoimmune pathology and a potential therapeutic target in MS [4].

Beyond MS, IL-6 has been implicated in other autoimmune and inflammatory conditions, including systemic lupus erythematosus (SLE) and neuromyelitis optica spectrum disorders (NMOSD), where IL-6 receptor blockade reduces relapse rates in the latter disease [5, 6]. Emerging data also associates peripheral blood IL-6 alterations with various neuropsychiatric conditions, underscoring its broader potential as a biomarker across central nervous system disorders [5]. Previous investigations into serum IL-6 in MS cohorts have yielded diverse results, highlighting the complexity of peripheral cytokine monitoring. While IL-6 is frequently elevated in the cerebrospinal fluid (CSF) during clinical relapses [7, 8], its presence in the serum is often lower or undetectable compared to central compartments [7]. Furthermore, recent studies in diverse populations have shown that while serum IL-6 can be elevated during relapses, it does not consistently correlate with all clinical subtypes [9].

Ocrelizumab (OCR) is a humanized monoclonal antibody that selectively depletes CD20^+^ B-cells and is effective in both Relapsing-Remitting (RRMS) and primary progressive MS (PPMS) [10]. Given IL-6’s role in B-cell function, it has been hypothesized that baseline IL-6 levels may predict disease activity or long-term outcomes in OCR-treated patients. However, existing data remains limited and inconclusive [11].

Therefore, the objective of this exploratory study was to investigate whether baseline and longitudinal serum IL-6 levels independently predict established clinical, radiological, and fluid markers of disease progression over 24 months in OCR-treated MS patients, and to compare these baseline levels with healthy controls.

## Methods

### Study population

**Fig. 1:**
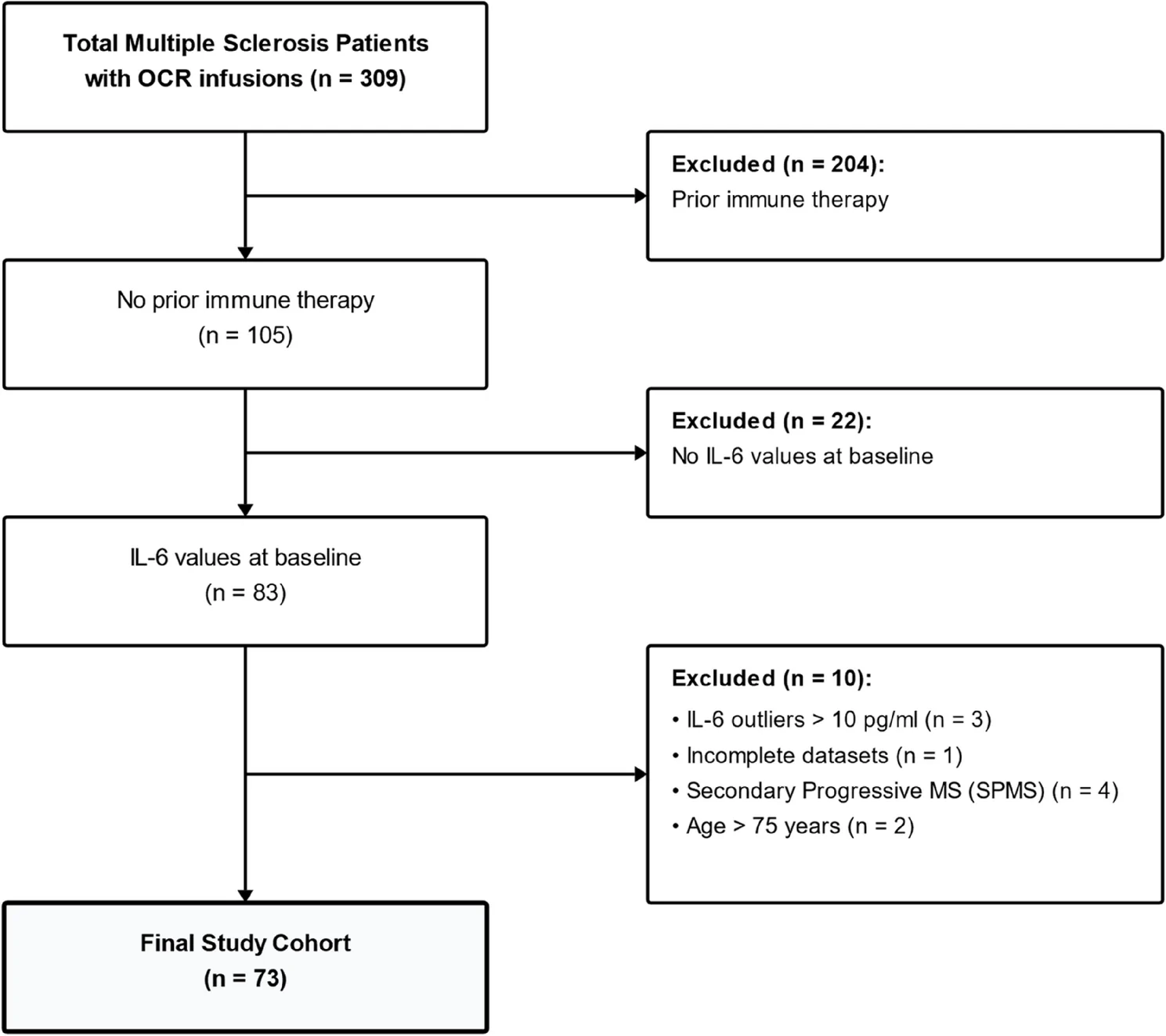
Patient cohort inclusion and exclusion flow diagram. A total of 309 multiple sclerosis (MS) patients receiving ocrelizumab (OCR) infusions were initially assessed for eligibility. Patients were sequentially excluded if they had a history of prior immune therapy (*n* = 204) or lacked baseline interleukin-6 (IL-6) measurements (*n* = 22). From the remaining 83 patients, an additional 10 were excluded due to baseline IL-6 outlier values (> 10 pg/ml; *n* = 3), incomplete clinical datasets (*n* = 1), a diagnosis of secondary progressive MS (SPMS; *n* = 4), or exceeding the upper age limit of 75 years (*n* = 2). The final cohort included in the analysis comprised 73 patients. *Abbreviations*: MS = multiple sclerosis; OCR = ocrelizumab; IL-6 = interleukin-6; SPMS = secondary progressive multiple sclerosis

This exploratory, single-center retrospective study used data from the MS database of the Department of Neurology, Heinrich-Heine-University Düsseldorf, Germany (2018–2025), including consecutively enrolled patients during the study period. Clinical data was recorded during routine visits every six months and made available for research after written informed consent. Only patients diagnosed with RRMS or PPMS according to the 2017 McDonald criteria were included. Additional inclusion criteria comprised: [1] initiation of ocrelizumab (OCR) therapy and [2] availability of baseline serum samples collected prior to the first OCR infusion. Patients who experienced a relapse within three months prior to OCR initiation or were unable to provide consent were excluded. To minimize confounding from acute inflammation, baseline C-reactive protein (CRP) levels and leukocyte counts were obtained. Included patients did not show evidence of active infection, defined as CRP above the reference range, leukocytosis, or clinical signs of infection at the time of sample collection. Additionally, Patients were excluded if they had a history of prior immune therapy for multiple sclerosis or lacked baseline interleukin-6 (IL-6) measurements. Patients with baseline IL-6 outlier values, incomplete clinical datasets, diagnosis of secondary progressive MS, or exceeding the upper age limit of 75 years were also excluded (Fig. 1). For comparison, baseline IL-6 levels from 86 healthy individuals without any history of autoimmune or chronic inflammatory disease were included as HC. This study was conducted in accordance with the STROBE (Strengthening the Reporting of Observational Studies in Epidemiology) guidelines for cohort studies [12].

Disability status and disease severity of MS patients were assessed using the Expanded Disability Status Scale (EDSS), a clinician-rated scale ranging from 0 (normal neurological examination) to 10 (death due to MS) [13] EDSS was recorded at baseline (within one month prior to treatment initiation) and every six months thereafter, with changes from baseline analyzed to evaluate disability progression.

### Outcome definitions

Disability progression and disease activity were defined using established clinical and radiological criteria See Table 1 for a summary of clinical outcome definitions, confirmation windows, and their respective statistical models. Confirmed Disability Accumulation (CDA) was defined as an increase in EDSS of ≥ 1.0 points for patients with baseline EDSS ≤ 5.5, or ≥ 0.5 points for baseline EDSS > 5.5, confirmed at a subsequent visit ≥ 12 weeks later. Progression Independent of Relapse Activity (PIRA) was defined as CDA occurring without a preceding relapse between the two relevant EDSS assessments or within the 12 weeks prior to baseline [14]. Relapse-Associated Worsening (RAW) was defined as CDA occurring during a relapse, with sustained disability progression persisting for ≥ 12 weeks; transient EDSS changes related to relapses without confirmation were excluded from both PIRA and RAW [15]. Relapses were defined as neurological deterioration lasting more than 24 h, unrelated to infection, and verified within 7 days. Absence of EDSS progression was defined as no confirmed disability increase over six months, with thresholds depending on baseline EDSS (0: ≥1.5 points; 1–5: ≥1 point; >5: ≥0.5 points). MRI activity was defined by new T1 gadolinium-enhancing lesions or new/enlarging T2 lesions in clinical routine measurements. No Evidence of Disease Activity-3 (NEDA-3) was defined as the absence of: [1] new or enlarging T2 lesions or T1 gadolinium-enhancing lesions on MRI [2], clinical relapses, and [3] confirmed disability accumulation (CDA) over the observation period. Conversely, loss of NEDA-3 was defined as the occurrence of any of these events. This composite outcome was assessed according to the standardized criteria previously described [16].

**Table 1:** Summary of clinical outcome definitions and applied statistical models. Overview of the clinical and radiological endpoints evaluated over the 24-month follow-up period, including specific confirmation windows for disability accumulation. The table also specifies the primary statistical models used to assess the predictive value of baseline biomarkers for each respective outcome

Clinical Endpoint	Definition	Confirmation Window	Statistical Model(s) Used
CDA (Confirmed Disability Accumulation)	Increase in EDSS of ≥ 1.0 points for patients with baseline EDSS ≤ 5.5, or ≥ 0.5 points for baseline EDSS > 5.5.	Confirmed at a subsequent visit ≥ 12 weeks later.	Cox proportional hazards models.
PIRA (Progression Independent of Relapse Activity)	CDA occurring without a preceding relapse between the two relevant EDSS assessments or within the 12 weeks prior to baseline.	Sustained for ≥ 12 weeks (inherent to the CDA definition).	Multivariable linear regression.
RAW (Relapse-Associated Worsening)	CDA occurring during a relapse.	Sustained disability progression persisting for ≥ 12 weeks.	Multivariable Cox regression^1^
MRI activity	New T1 gadolinium-enhancing lesions or new/enlarging T2 lesions in clinical routine measurements.	Follow-up clinical routine measurements.	Logistic regression.
NEDA-3 (No Evidence of Disease Activity-3)	The absence of: (1) new/enlarging T2 or T1 gadolinium-enhancing lesions, (2) clinical relapses, and (3) CDA.	Over the observation period (24 months).	Cox proportional hazards models, multivariable linear regression, or logistic regression.
Loss of NEDA-3	The occurrence of any of the events listed under NEDA-3 (MRI activity, relapse, or CDA).	Over the observation period (24 months).	Cox proportional hazards models, multivariable linear regression, or logistic regression. Between-group comparisons used paired t-tests or Wilcoxon signed-rank tests.

*Abbreviations:* *CDA* Confirmed Disability Accumulation, *MRI* Magnetic Resonance Imaging, *NEDA-3* No Evidence of Disease Activity-3, *PIRA* Progression Independent of Relapse Activity, *RAW* Relapse-Associated Worsening

^*1*^Not performed due to the limited number of events, *n* = 3)

### Laboratory measures

Blood samples were obtained from patients with MS at baseline and every six months for up to two years, scheduled within two weeks prior to each subsequent OCR infusion; HCs were sampled at baseline only. Blood was drawn via a short catheter from an antecubital vein into Monovettes (Sarstedt, Nümbrecht, Germany). Serum tubes were allowed to clot for 30–60 min, centrifuged at 3000 rpm for 10 min, aliquoted, and initially frozen at − 20 °C. Within four weeks, samples were transferred to a − 80 °C freezer for long-term storage until final analysis. EDTA tubes were used for leukocyte counts, which were analyzed directly without centrifugation.

Serum was used for C‑reactive protein (CRP), immunoglobulin G (IgG), immunoglobulin A (IgA), immunoglobulin M (IgM), serum neurofilament light chain (sNfL), serum glial fibrillary acidic protein (sGFAP), and IL‑6. All biomarkers were assessed at baseline, whereas IL‑6 was additionally measured at six-month intervals throughout the study period. Quantification of serum IL-6, sNfL, and sGFAP was performed at the Central Institute for Clinical Chemistry and Laboratory Diagnostics, Medical Faculty, University Hospital Düsseldorf, Germany. All three biomarkers were analyzed using electrochemiluminescence immunoassays (ECLIA) on the Roche Cobas 8000 analyzer (Elecsys^®^ module) according to the manufacturer’s instructions. Serum IL-6 was quantified using accredited routine diagnostic procedures according to DIN EN ISO 15,689. The IL-6 assay had a lower limit of quantification (LLOQ) of 1.5 pg/mL and an inter-assay coefficient of variation (intermediate precision) of < 5.2% at low concentrations (3.47 pg/mL). Values below the LLOQ were handled as 1.4 pg/mL. Laboratory personnel were blinded to clinical data. Furthermore, clinical raters assessing EDSS and radiologists evaluating MRI were fully blinded to the laboratory biomarker results. Z-scores for sNfL and sGFAP were calculated based on previously published methods [17, 18].

### Statistical analysis

All analyses were conducted in RStudio. Demographic, clinical, and biomarker data were summarized using descriptive statistics. Normal distribution was tested using the Shapiro–Wilk test. Non-normally distributed data were presented as median and interquartile range (IQR), whereas normally distributed data were reported as mean ± standard deviation (SD). Between-group comparisons of continuous variables were performed using Welch’s t-test for normally distributed data or the Wilcoxon rank-sum test for non-normal data. Paired longitudinal comparisons within groups were conducted using paired t-tests or Wilcoxon signed-rank tests, as appropriate. Associations between baseline biomarkers and clinical outcomes, including CDA, PIRA, RAW, NEDA-3, loss of NEDA-3, and MRI activity, were assessed using Cox proportional hazards models, multivariable linear regression, or logistic regression. Cox proportional hazards models, multivariable linear regression and logistic regression were adjusted for age, sex and baseline EDSS. The proportional hazards assumption for Cox models was evaluated using Schoenfeld residuals and no violations were observed. Missing data in longitudinal analyses were handled via complete case analysis. Rank-based linear models were applied for comparisons of IL-6 levels across MS subtypes and over time. The dichotomization of IL-6 at the median for Kaplan-Meier survival analysis was utilized as an exploratory, secondary approach to visualize the data. A p-value < 0.05 was considered statistically significant, with correction for multiple comparisons applied where relevant.

### Approvals, registrations, and consent

The study was approved by the Ethics Committee of Heinrich Heine University (study number: 5951R) and conducted in accordance with the Declaration of Helsinki. All participants provided informed consent. Clinical trial number: not applicable.

### Data availability

The datasets analyzed during the current study are available from the corresponding author on reasonable request.

## Results

### Baseline clinical and biomarker characteristics

**Fig. 2:**
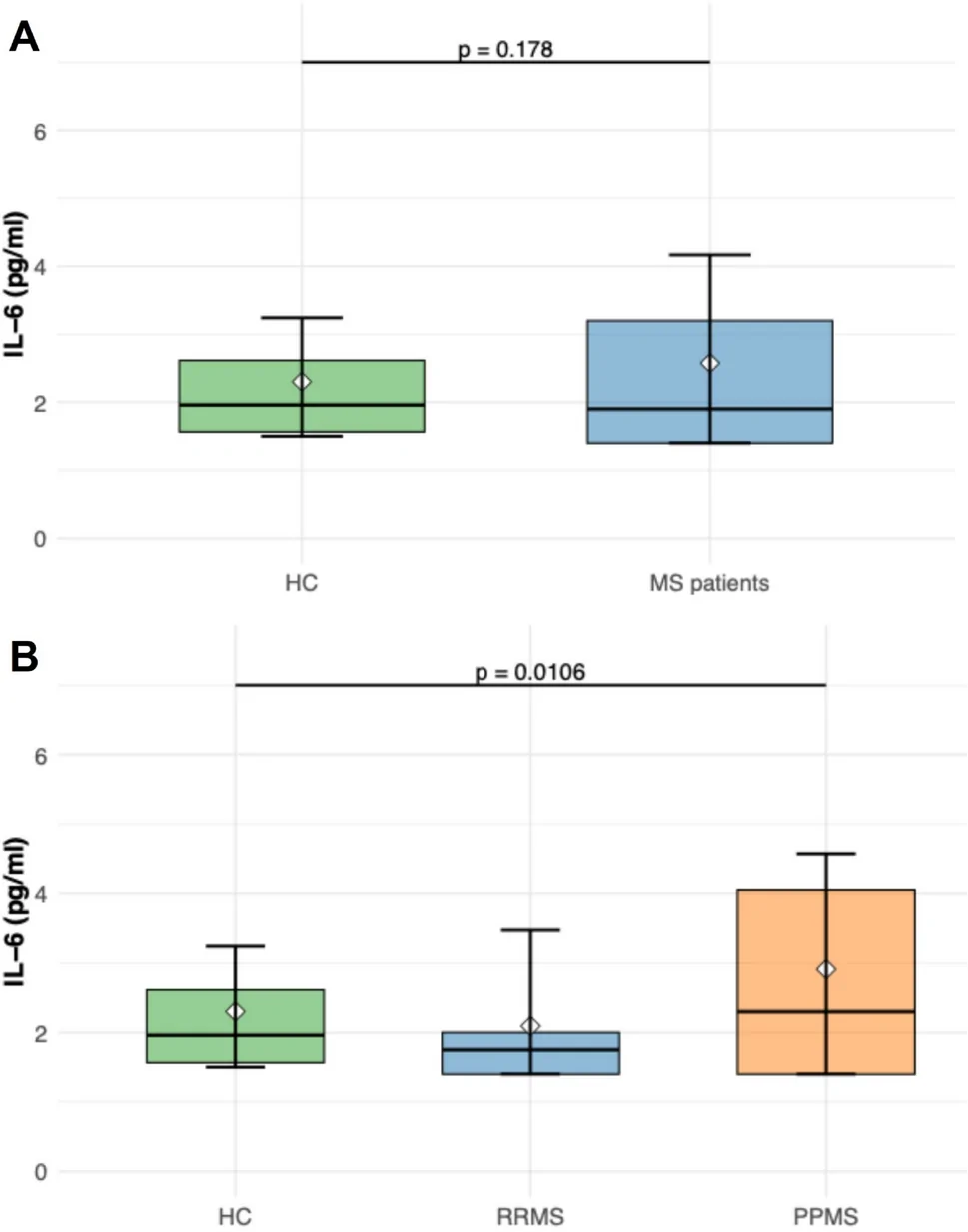
Comparison of baseline serum interleukin-6 (IL-6) levels between groups. **A **Comparison between the total MS cohort and HC. No significant difference was observed (*p* = 0.178, rank-based linear model adjusted for age and sex). **B **Subgroup analysis of IL-6 levels across RRMS, PPMS, and HC. While an overall group effect was noted (*p* = 0.0106, ANOVA), post-hoc pairwise comparisons did not reveal significant differences between individual groups. *Abbreviations:* HC: Healthy Controls; IL-6: Interleukin-6; MS: Multiple Sclerosis; PPMS: Primary Progressive Multiple Sclerosis; RRMS: Relapsing-Remitting Multiple Sclerosis

A total of 73 MS patients were included, comprising 43 with PPMS (58.9%) and 30 with RRMS (41.1%) (Fig. 1). Baseline characteristics are presented in Tables 2 and 3. The mean age of the entire MS cohort was 52.2 ± 12.7 (SD) years, and 54.8% were female, with PPMS patients being older than those with RRMS (58.3 ± 9.3 vs. 43.5 ± 12 years, *p* < 0.001) and showing a slight male predominance (58.1% vs. 50%, *p* = 0.654). HC (*n* = 86) had a mean age of 46.1 ± 11.8 years and were in 72.1% female. Compared with MS patients HC were significantly younger (*p* < 0.001). Median disease duration was significantly longer in PPMS (115 months, IQR 78) compared with RRMS (73 months, IQR 48; *p* < 0.001). Baseline EDSS was also higher in PPMS (median 4.0, IQR 3.375) than in RRMS (median 2.0, IQR 2.5; *p* < 0.001) (Table 2.2). Baseline IL-6 levels were similar between the overall MS cohort and HC (MS: 1.90 [IQR 1.8] pg/mL vs. HC: 1.96 [IQR 1.1] pg/mL; rank-based linear model adjusted for age and sex; *p* = 0.178) (Fig. 2A). Subgroup analysis by MS type revealed that baseline mean IL-6 level was higher in PPMS (2.30 [IQR 2.7] pg/mL), compared to RRMS patients (1.75 [IQR 0.6] pg/mL; *p* = 0.024). A significant overall group effect was observed (ANOVA; *p* = 0.011); however, post-hoc pairwise comparisons adjusted for multiple testing did not reveal statistically significant differences between individual groups, although a trend toward lower IL-6 levels in RRMS compared with HC was evident (Fig. 2B).

**Table 2:** Baseline characteristics of the MS cohort and healthy controls. Demographic, clinical, and laboratory parameters are compared between the total multiple sclerosis (MS) cohort (n=73) and healthy controls (HC, n=86) prior to ocrelizumab initiation.

Variable	Total MS Cohort (n=73)	HC (n=86)	p-value
Age (years, mean ± SD)	52.2 ± 12.7	46.1 ± 11.8	<0.001
Female sex (n, %)	40 (54.8%)	62 (72.1%)	0.036
Months since disease manifestation (median; range; IQR)	91 (16–427; IQR 71)	NA	NA
EDSS (median; range; IQR)	3 (0–7; IQR 3.3)	NA	NA
Relapse within 24M (n, %)	6 (7.8%)	NA	NA
CDA within 24M (n, %)	22 (30.1%)	NA	NA
PIRA within 24M (n, %)	20 (27.4%)	NA	NA
RAW within 24M (n, %)	2 (2.7%)	NA	NA
Loss of NEDA-3 within 24M (n, %)	31 (42.5%)	NA	NA
IL-6 [pg/ml] (median; IQR)	1.90 (IQR 1.8)	1.96 (IQR 1.1)	0.334
sNfL z-score (median; IQR)	-0.10 (IQR 2.6)	NA	NA
sGFAP z-score (median; IQR)	0.28 (IQR 1.5)	NA	NA
CRP [mg/L] (median; IQR)	0.09 (IQR 0.2)	0.07 (IQR 0.1)	<0.001
IgG [mg/dl] (median; IQR)	952 (IQR 348.5)	885 (IQR 250)	0.761
IgM [mg/dl] (median; IQR)	86 (IQR 58.5)	NA	NA
Vitamin D [ng/ml] (median; IQR)	23.0 (IQR 19)	NA	NA

Continuous variables are presented as mean ± SD or median (range; IQR). Categorical variables are presented as n (%). P-values represent comparisons between the total MS cohort and HC using Welch’s t-test or Wilcoxon rank-sum test

*Abbreviations:* *CDA *Confirmed Disability Accumulation, *CRP *C-reactive Protein, *EDSS *Expanded Disability Status Scale, *HC *Healthy Controls, *IQR *Interquartile Range, *NEDA-3 *No Evidence of Disease Activity-3, *MS *Multiple Sclerosis, *NA *Not Applicable, *PIRA *Progression Independent of Relapse Activity, *RAW *Relapse-Associated Worsening, *SD *Standard Deviation, *sGFAP *Serum Glial Fibrillary Acidic Protein, *sNfL *Serum Neurofilament Light Chain

While baseline sGFAP z-scores were similar between MS subtypes, median baseline sNfL z-scores were elevated in RRMS (2.12 [IQR 1.9]) compared with PPMS (− 0.41 [IQR 1.6]; *p* < 0.001) (Suppl. Figure 1). CRP levels were slightly higher in PPMS compared to healthy controls, though all retained samples were below the clinical threshold for acute infection (Suppl. Figure 2).

During the 24-month follow-up, 22 MS patients (30.1%) experienced CDA, 20 experienced PIRA (27.4%), and 2 experienced RAW (2.7%), with loss of NEDA-3 observed in 31 of 73 patients (42.5%). See Supplementary Table 1 for baseline characteristics stratified by maintenance versus loss of NEDA-3, and Supplementary Table 2 for baseline characteristics comparing IL-6 High vs. IL-6 Low groups.

### Predictive value of baseline biomarkers

**Fig. 3:**
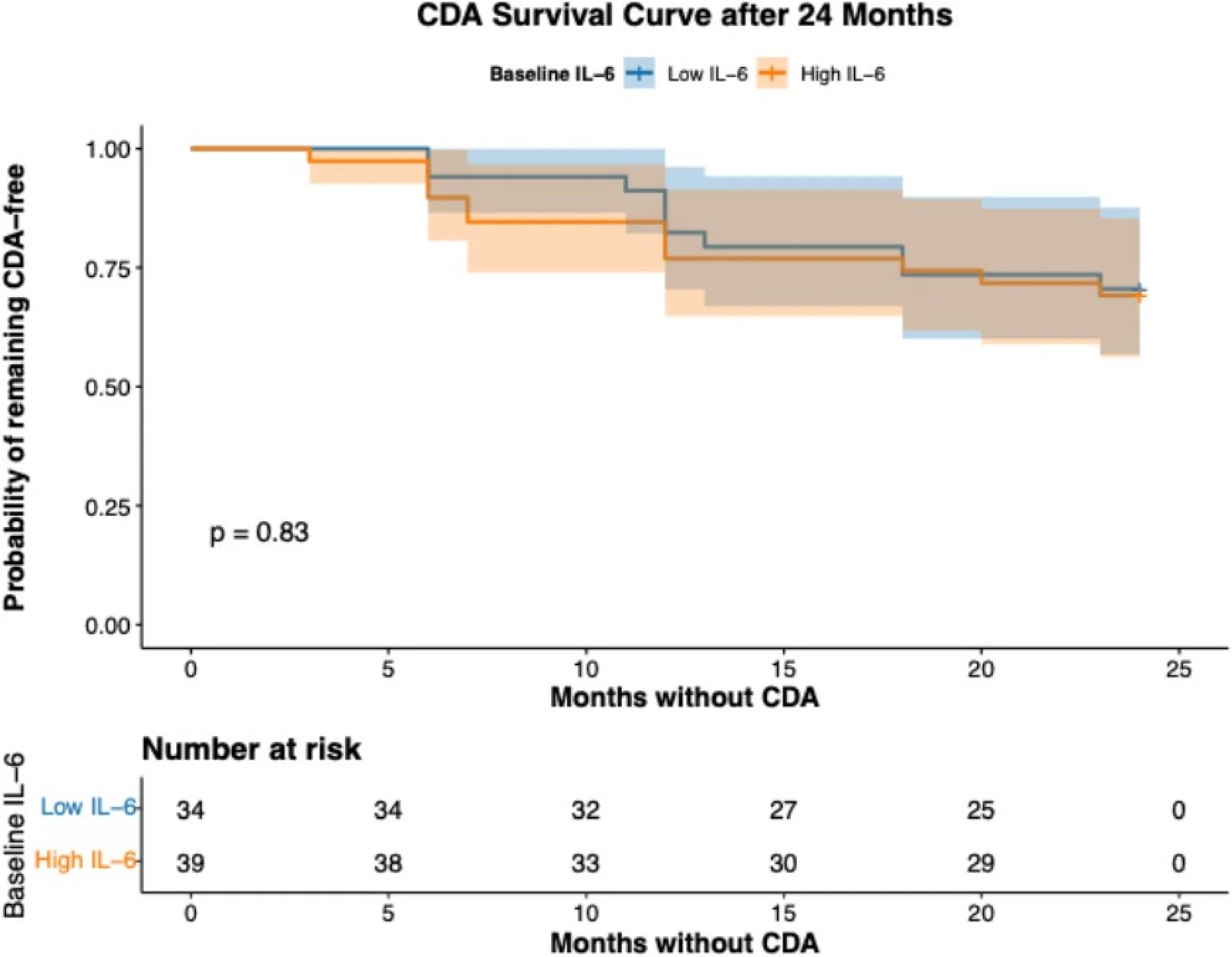
Time to CDA over 24 months stratified by baseline IL-6. Kaplan-Meier survival analysis showing the probability of remaining free from CDA. Patients were dichotomized into “High” and “Low” groups based on the median baseline IL-6 level. No significant difference in CDA risk was observed between the two groups (HR = 1.83; 95% CI: 0.73–4.61; p-value = 0.989). *Abbreviations:* CDA: Confirmed Disability Accumulation; CI: Confidence Interval; HR: Hazard Ratio; IL-6: Interleukin-6

Cox proportional hazard models revealed no significant associations between baseline IL-6 and occurrence of CDA (Suppl. Table 3). In an exploratory, secondary approach utilizing dichotomization of IL-6 at the median, there was no statistically significant association with the risk of CDA (HR 1.83; 95% CI 0.73–4.61; p_adj_ = 0.989) (Fig. 3).

**Table 3:** Baseline characteristics of PPMS and RRMS patients. Baseline clinical, demographic, and biomarker profiles are stratified by multiple sclerosis subtype, comparing primary progressive MS (PPMS, n=43) with relapsing-remitting MS (RRMS, n=30).

Variable	PPMS (n=43)	RRMS (n=30)	p-value
Age (years, mean ± SD)	58.3 ± 9.3	43.5 ± 12	<0.001
Female sex (n, %)	25 (58.1%)	15 (50%)	0.654
Months since disease manifestation (median; range; IQR)	115 (16–427; IQR 78)	73 (37–415; IQR 48)	<0.001
EDSS (median; range; IQR)	4 (1.5–7; IQR 3.4)	2 (0.0–6.5; IQR 2.5)	<0.001
Relapse within 24M (n, %)	NA	6 (20%)	NA
CDA within 24M (n, %)	18 (41.9%)	4 (13.3%)	0.019
PIRA within 24M (n, %)	18 (41.9%)	2 (6.7%)	0.002
RAW within 24M (n, %)	NA	2 (8%)	NA
Loss of NEDA-3 within 24M (n, %)	20 (46.5%)	11 (36.7%)	0.551
IL-6 [pg/ml] (median; IQR)	2.30 (IQR 2.7)	1.75 (IQR 0.6)	0.024
sNfL z-score (median; IQR)	-0.41 (IQR 1.6)	2.12 (IQR 1.9)	0.005
sGFAP z-score (median; IQR)	0.28 (IQR 1.1)	0.62 (IQR 2.6)	0.806
CRP [mg/L] (median; IQR)	0.10 (IQR 0.3)	0.09 (IQR 0.11)	0.341
IgG [mg/dl] (median; IQR)	969 (IQR 225)	829 (IQR 459)	0.018
IgM [mg/dl] (median; IQR)	87 (IQR 62)	82 (IQR 68)	0.153
Vitamin D [ng/ml] (median; IQR)	22.0 (IQR 20)	23.5 (IQR 16.5)	0.742

Characteristics are stratified by Multiple Sclerosis subtype. P-values represent comparisons between PPMS and RRMS groups using Welch’s t-test or Wilcoxon rank-sum test

*Abbreviations:* *CDA *Confirmed Disability Accumulation, *CRP *C-reactive Protein, *EDSS *Expanded Disability Status Scale, *IQR *Interquartile Range, *NEDA-3 *No Evidence of Disease Activity-3, *NA *Not Applicable, *PIRA *Progression Independent of Relapse Activity, *PPMS *Primary Progressive Multiple Sclerosis, *RAW *Relapse-Associated Worsening, *RRMS *Relapsing-Remitting Multiple Sclerosis, *SD *Standard Deviation, *sGFAP *Serum Glial Fibrillary Acidic Protein, *sNfL *Serum Neurofilament Light Chain

In RRMS, baseline IL-6 was not associated with relapse occurrence over two years (median 1.75 vs. 1.8 pg/mL; rank-based linear model adjusted for age and sex; *p* = 0.913). Multivariable Cox regression for RAW was not performed due to the limited number of events (*n* = 2).

Multivariable linear regression identified baseline EDSS as the sole predictor of EDSS at 24 months (β = 0.74; 95% CI 0.41–1.08; *p* = 0.005), while no other biomarkers, including sNfL and sGFAP z-scores and baseline IL-6, were associated with final disability accrual or PIRA. Furthermore, the inclusion of baseline IL-6 did not meaningfully improve the prognostic prediction models beyond the variance already explained by sNfL and sGFAP alone.

Similarly, logistic regression demonstrated no relationship between baseline IL-6 and new T2 lesions on follow-up MRI (OR 0.86; 95% CI 0.53–1.39; *p* = 0.53), with subgroup analyses in RRMS (OR 0.27; 95% CI 0.01–5.21; *p* = 0.38) and PPMS (OR 1.04; 95% CI 0.61–1.77; *p* = 0.88) showing consistent findings.

### Longitudinal biomarker dynamics

**Fig. 4:**
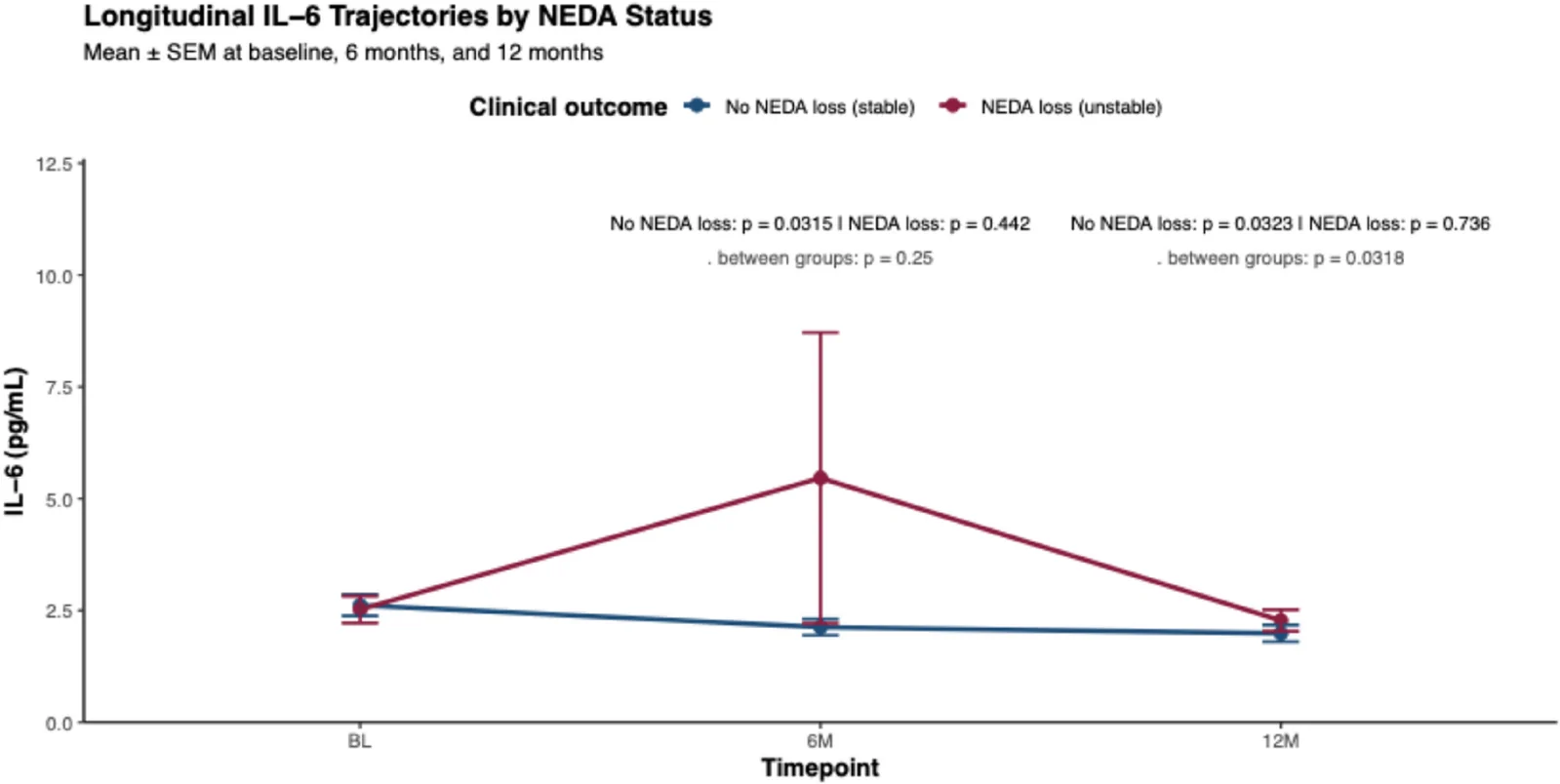
Longitudinal change in serum IL-6 levels at 6 and 12 months by loss of NEDA-3 status. Boxplots represent the mean change in IL-6 concentrations from baseline to 12 months post-ocrelizumab initiation. Patients maintaining NEDA-3 status exhibited a significant reduction in IL-6 (mean − 1.30 pg/mL), whereas those with loss of NEDA-3 remained stable (mean 0.09 pg/mL). The between-group difference was statistically significant (*p* = 0.0318), suggesting a potential association between IL-6 reduction and sustained clinical stability. *Abbreviations:* BL: Baseline value; 6M: 6 months after baseline; 12M: 12 months after baseline; IL-6: Interleukin-6; NEDA: No Evidence of Disease Activity-3

Longitudinal analysis of serum IL-6 levels of 25 patients over 12 months during B-cell depleting therapy revealed largely stable concentrations, with no significant differences observed at any follow-up compared to baseline. This stability persisted across MS subtypes (RRMS and PPMS). Upon stratification by clinical response, early IL-6 dynamics between baseline and 6 months did not differ significantly between patients maintaining NEDA-3 and those with loss of NEDA-3 (*p* = 0.126). However, by 12 months post-ocrelizumab initiation, patients maintaining NEDA-3 exhibited a mean decrease in IL-6 from baseline to 12 months (-1.30 pg/mL), whereas patients with disease activity showed no significant change (0.09 pg/mL). The between-group difference in IL-6 change over 12 months was statistically significant (*p* = 0.0318) (Fig. 4). The between-group difference in IL-6 changes from baseline to 12 months indicated a trend toward significance after adjustment for multiple comparisons (*p* = 0.064). No immediate temporal correlation was found between the exact timing of a relapse and a spike in IL-6, though the limited number of RAW events (*n* = 3) restricted this analysis.

## Discussion

In this exploratory retrospective cohort of 73 MS patients, we found that while mean baseline IL-6 was higher in PPMS, baseline serum IL-6 levels did not independently predict PIRA, RAW, CDA, or MRI activity over 24 months of ocrelizumab therapy after adjusting for covariates. However, we observed that patients who maintained NEDA-3 status experienced a significant 50% reduction in longitudinal IL-6 levels, a change not seen in those with disease activity.

Ocrelizumab therapy was intentionally incorporated in our cohort to investigate biomarkers predictive of disease activity in both PPMS and RRMS patients. The rationale for this investigation lies in the complex interplay between B cells and IL-6. IL-6-producing B-cells drive proinflammatory Th17 responses integral to MS pathogenesis, and ocrelizumab effectively targets these cells, thereby reducing inflammation and relapses [19].

Hypothetically, the depletion of these CD20^+^ B-cells should result in a concurrent reduction of serum IL-6, serving as a marker for therapeutic efficacy. However, despite effective B-cell depletion, a subset of patients continued to experience disease activity, indicating that compensatory or IL-6-independent pathways underpin progression in these cases [20]. The observed longitudinal decline in IL-6 among patients maintaining NEDA-3 may directly reflect the immunomodulatory effects of sustained B-cell depletion, as IL-6-producing B-cells drive proinflammatory Th17 responses [19]. Conversely, stable IL-6 levels in patients with ongoing disease activity highlight the potential need for composite biomarker approaches that integrate inflammatory markers (IL-6) with indices of neuroaxonal injury and glial activation (sNfL, sGFAP) to better capture the multifaceted immunopathology of MS. Within this context, our findings indicate that serum IL-6 alone lacks sufficient sensitivity to predict clinical disease activity or associated neurodegenerative markers.

Although serum IL-6 did not differentiate MS patients from controls, mean baseline levels were highest in PPMS, suggesting a possible association with progressive disease. This pattern aligns with the concept that PPMS is characterized by chronic, low-grade inflammation and neurodegeneration, processes known to drive increased systemic IL-6 production, particularly with advancing age. Notably, previous transcriptomic studies have reported reduced IL-6 receptor expression in PPMS compared with RRMS, a finding that may reflect receptor downregulation or shedding in the setting of chronically elevated IL-6 levels [21].

The higher baseline IL-6 in PPMS patients might also reflect age-dependent immune signatures, as our PPMS cohort trended towards an older age. This chronic low-grade systemic inflammation may contribute to the relatively high rate of loss of NEDA-3 (42.5%) and CDA observed in this real-world cohort [22].

Taken together, higher IL-6 concentrations in PPMS combined with lower IL-6R expression may represent a compensatory or exhaustion-related immune signature rather than an acute inflammatory response. Furthermore, this may indicate that elevated IL-6 is more characteristic of PPMS and could help distinguish disease courses, though the small sample size limits firm conclusions.

Notably, sNfL and GFAP z-scores in this cohort did not predict outcomes, in contrast to other studies [17, 23], which may be explained by the higher age of our patients and the relatively low relapse activity.

In our subgroup with available longitudinal serum samples, IL-6 levels remained largely stable over 24 months of B-cell depleting therapy. When patients were stratified by NEDA-3 status at 12 months, those who maintained NEDA-3 showed significant reductions in IL-6, whereas patients experiencing loss of NEDA-3 exhibited minimal change. The between-group difference at 12 months suggested a trend toward significance after correction for multiple testing, although sample sizes were limited. These observations raise the possibility that longitudinal IL-6 dynamics may reflect clinical stability under anti-CD20 therapy, warranting further investigation in larger cohorts to determine its potential utility as a biomarker.

Notably, prior research consistently links cerebrospinal fluid (CSF) IL-6 - not serum IL-6 - with MS disease activity and disability. Elevated CSF IL-6 correlates with severity and is higher in PPMS compared with controls. This divergence likely reflects cytokine compartmentalization and blood-brain barrier selectivity limiting IL-6 spill-over into serum, except possibly in progressive MS with chronic blood-brain barrier disruption [24]. Our lack of CSF IL-6 data notwithstanding, this mechanistic insight contextualizes our negative serum IL-6 findings, underscoring CSF IL-6 as a more sensitive CNS inflammation biomarker.

Thus, serum IL-6 alone cannot capture the full spectrum of inflammatory and neurodegenerative processes in MS progression under B-cell therapy. Research should emphasize the multifaceted B-cell - IL-6 axis and develop composite biomarkers covering diverse pathophysiological routes, improving patient stratification and refining therapeutic targeting beyond IL-6.

Comparatively, IL-6 is a robust biomarker in neuromyelitis optica spectrum disorder (NMOSD), where it closely correlates with disease activity, relapse severity, and disability both in serum and CSF. IL-6 drives NMOSD pathogenesis by promoting plasmablast survival, aquaporin-4 antibody production, blood-brain barrier disruption, and enhanced proinflammatory T-cell activation. IL-6 receptor blockade significantly reduces NMOSD relapses, underscoring IL-6’s role as both biomarker and therapeutic target [25]. In contrast, MS exhibits heterogeneous immunopathology with multiple cytokines and mechanisms fluctuating by stage and subtype, limiting IL-6’s predictive value. This disease-specific distinction emphasizes the importance of appropriate biomarker validation tailored to distinct CNS autoimmune disorders.

### Limitations

Our study has several limitations. First, the exploratory, retrospective, single-center design and moderate sample size (*n* = 73) limit statistical power and generalizability. Therefore, the possibility of Type II errors regarding the predictive value of IL-6 cannot be excluded. Second, while we controlled for overt acute infections, potential residual confounding from subclinical infections or unrecorded systemic comorbidities known to influence IL-6 could be present. Finally, single baseline measures may miss post-treatment cytokine dynamics, and missing data in our longitudinal sampling may introduce bias.

## Conclusion

In conclusion, our data reinforce IL-6’s limited utility as a serum biomarker for disease monitoring in B-cell–treated MS. However, the significant longitudinal decline specifically in stable patients suggests that monitoring IL-6 dynamics within a multiparametric framework—rather than relying on static baseline measures—may provide clinically meaningful information regarding sustained therapeutic response. Future studies should integrate longitudinal, multiparametric approaches combining cytokine profiles, neuroaxonal injury markers, glial activation, and advanced imaging across larger, balanced cohorts to clarify IL-6’s combined biomarker potential within the broader inflammatory milieu governing MS progression and therapy response. This will facilitate individualized disease monitoring and precision therapeutic strategies.

## Supplementary Information

Supplementary Material 1.

## Data Availability

The datasets analyzed during the current study are available from the corresponding author on reasonable request.

## Abbreviations

CDA: Confirmed Disability Accumulation
CRP: C-reactive Protein
CSF: Cerebrospinal Fluid
ECLIA: Electrochemiluminescence Immunoassay
EDSS: Expanded Disability Status Scale
HC: Healthy Controls
IgA: Immunoglobulin A
IgG: Immunoglobulin G
IgM: Immunoglobulin M
IL-6: Interleukin-6
IQR: Interquartile Range
MRI: Magnetic Resonance Imaging
MS: Multiple Sclerosis
NEDA-3: No Evidence of Disease Activity-3
NMOSD: Neuromyelitis Optica Spectrum Disorders
OCR: Ocrelizumab
PIRA: Progression Independent of Relapse Activity
PPMS: Primary Progressive Multiple Sclerosis
RAW: Relapse-Associated Worsening
RRMS: Relapsing–Remitting Multiple Sclerosis
SD: Standard Deviation
sGFAP: Serum Glial Fibrillary Acidic Protein
SLE: Systemic Lupus Erythematosus
sNfL: Serum Neurofilament Light Chain
NA: Not Applicable
