# Supplementary material for: Investigating the role of serum IL-6 in predicting outcomes of b-cell depleting therapy in multiple sclerosis: a retrospective cohort study
Source: BMC Neurol. 2026 Aug 21;26:536. doi: 10.1186/s12883-026-05252-2 (PMC13495299; doi:10.1186/s12883-026-05252-2)
Supplement: Supplementary file 1 [file 12883_2026_5252_MOESM1_ESM.docx]

**Supplementary Data**

**Supplementary Table 1:** Baseline characteristics of patients stratified by NEDA-3 status at 24 months. Comparison of baseline demographic, clinical, and laboratory parameters between patients who maintained No Evidence of Disease Activity-3 (NEDA-3) and those who experienced a loss of NEDA-3 over the 24-month observation period. Continuous variables are presented as mean ± SD or median (IQR). Categorical variables are presented as n (%). P-values reflect between-group comparisons using Welch’s t-test, Wilcoxon rank-sum test, or Fisher’s exact test to assess baseline differences driving treatment response.

|  | **Conserved NEDA-3 status (n=32)** | **Loss of NEDA-3 status (n=30)** | **p-value** |
| --- | --- | --- | --- |
| **Age at BL** (years) | 51.2 ± 14.3 | 49.8 ± 13.7 | 0.692 |
| **Female sex** | 17 (53.1%) | 18 (60.0%) | 0.618 |
| **EDSS at BL** | 3.0 (2.0 - 5.0) | 3.0 (2.0 - 4.1) | 0.735 |
| **No. RRMS patients** | 18 (56.3%) | 17 (56.7%) | 1.000 |
| **No. PPMS patients** | 14 (43.8%) | 13 (43.3%) | 1.000 |
| **Relapse in 12 months** | 1.0 (0.0 - 2.0) | 1.0 (0.0 - 1.8) | 0.840 |
| **PIRA within 12 months** | 0 (0.0%) | 15 (50.0%) | NA |
| **RAW within 12 months** | 0 (0.0%) | 5 (16.7%) | NA |
| **IL-6 at BL** (pg/ml) | 1.90 (1.40 - 3.05) | 1.90 (1.40 - 3.28) | 0.970 |
| **IL-6 at 6 months** (pg/ml) | 1.83 (1.49 - 2.44) | 2.03 (1.67 - 2.85) | 0.516 |
| **IL-6 at 12 months** (pg/ml) | 1.40 (1.40 - 1.40) | 2.16 (1.40 - 2.74) | 0.109 |
| *Abbreviations:* BL: Baseline; EDSS: Expanded Disability Status Scale; IQR: Interquartile Range; PIRA: Progression Independent of Relapse Activity; PPMS: Primary Progressive Multiple Sclerosis; RAW: Relapse-Associated Worsening; RRMS: Relapsing-Remitting Multiple Sclerosis; SD: Standard Deviation. | | | |

**Supplementary Table 2:** Baseline demographic, clinical, and biomarker characteristics stratified by median IL-6 levels. Comparison of baseline characteristics between patients with "High IL-6" and "Low IL-6" levels, categorized based on the median baseline serum IL-6 concentration of the total multiple sclerosis cohort (1.90 pg/mL). Continuous variables are presented as median (interquartile range). Categorical variables are presented as n (%). P-values represent between-group comparisons using Welch’s t-test, Wilcoxon rank-sum test, or Fisher’s exact test, as appropriate, to assess potential confounding imbalances.

|  | **IL-6 low (n=35)** | **IL-6 high (n=38)** | **p-value** |
| --- | --- | --- | --- |
| **Age at BL** (years) | 49.8 ± 13.5 | 58.7 ± 11.4 | 0.003 |
| **Female sex** | 17 (48.6%) | 23 (60.5%) | 0.352 |
| **EDSS at BL** | 3.0 (2.0 - 5.0) | 4.0 (2.2 - 5.8) | 0.129 |
| **No. RRMS patients** | 19 (54.3%) | 12 (31.6%) | 0.061 |
| **No. PPMS patients** | 16 (45.7%) | 26 (68.4%) | 0.061 |
| **Relapse in 12 months** | 1.0 (0.0 - 1.0) | 0.0 (0.0 - 1.8) | 0.504 |
| **PIRA within 12 months** | 11 (31.4%) | 13 (34.2%) | 1.000 |
| **RAW within 12 months** | 3 (8.6%) | 1 (2.6%) | 0.344 |
| **IL-6 at BL** (pg/ml) | 1.40 (1.40 - 1.75) | 3.20 (2.60 - 4.38) | NA |
| **IL-6 at 6 months** (pg/ml) | 2.12 (1.85 - 2.38) | 1.91 (1.70 - 2.71) | 0.932 |
| **IL-6 at 12 months** (pg/ml) | 1.40 (1.40 - 1.83) | 2.40 (1.78 - 3.05) | 0.102 |
| *Abbreviations:* BL: Baseline; EDSS: Expanded Disability Status Scale; IQR: Interquartile Range; PIRA: Progression Independent of Relapse Activity; PPMS: Primary Progressive Multiple Sclerosis; RAW: Relapse-Associated Worsening; RRMS: Relapsing-Remitting Multiple Sclerosis; SD: Standard Deviation. | | | |

**Supplementary Table 3:** Patient characteristics by CDA occurrence in those with longitudinal IL-6 data over 12 months. This subgroup includes patients with available serum samples at baseline, 6 months (V6), and 12 months (V12). No significant differences in baseline or longitudinal IL-6 levels were found between those who experienced CDA and those who did not.

|  | **Patients without CDA (n=13)** | **Patients with CDA (n=12)** | **p-value** |
| --- | --- | --- | --- |
| **Age at BL (years, mean ± SD)** | 56.1 ± 8.41 | 54.6 ± 12.8 | 0.73 |
| **Female sex (n, %)** | 7 (28%) | 6 (24%) | 1.0 |
| **EDSS at BL (median; IQR)** | 3.00 (IQR 2.5) | 3.50 (IQR 2.5) | 0.56 |
| **No. RRMS patients** | 6 (24%) | 3 (12%) | 0.41 |
| **No. PPMS patients** | 7 (28%) | 9 (36%) | 0.41 |
| **Relapse within 12M (n, %)** | NA | 2 (8%) | NA |
| **PIRA within 12M (n, %)** | NA | 10 (40%) | NA |
| **RAW within 12M (n, %)** | NA | 2 (8%) | NA |
| **IL-6 at BL [pg/ml] (median; IQR)** | 2.2 (IQR 2.1) | 1.9 (IQR 1.5) | 0.91 |
| **IL-6 at V6 [pg/ml] (median; IQR)** | 1.9 (IQR 0.802) | 2.64 (IQR 1.32) | 0.65 |
| **IL-6 at V12 [pg/ml] (median; IQR)** | 1.59 (IQR 0.51) | 2.33 (IQR 0.893) | 0.23 |
| *Abbreviations:* BL: Baseline; CDA: Confirmed Disability Accumulation; EDSS: Expanded Disability Status Scale; IQR: Interquartile Range; PIRA: Progression Independent of Relapse Activity; PPMS: Primary Progressive Multiple Sclerosis; RAW: Relapse-Associated Worsening; RRMS: Relapsing-Remitting Multiple Sclerosis; SD: Standard Deviation. | | | |

**Supplementary Figure 1:** Comparison of Baseline sNfL Z-scores Between RRMS and PPMS. Median baseline sNfL z-scores were significantly elevated in RRMS (2.12; IQR 1.9) compared with PPMS (-0.41; IQR 1.6; p = 0.00002).

*Abbreviations:* IQR: Interquartile Range; PPMS: Primary Progressive Multiple Sclerosis; RRMS: Relapsing-Remitting Multiple Sclerosis; sNfL: Serum Neurofilament Light Chain.

**Supplementary Figure 2:** Comparison of baseline C-reactive protein (CRP) levels. Comparison of CRP levels across RRMS, PPMS, and HC. While PPMS patients showed slightly higher levels compared to HC, all values remained below the clinical threshold for acute infection, minimizing confounding of IL-6 measurements.

*Abbreviations:* CRP: C-reactive Protein; HC: Healthy Controls; PPMS: Primary Progressive Multiple Sclerosis; RRMS: Relapsing-Remitting Multiple Sclerosis.
